# Mechanistic Insights into Eosinophilic Esophagitis: Therapies Targeting Pathophysiological Mechanisms

**DOI:** 10.3390/cells12202473

**Published:** 2023-10-18

**Authors:** Sara Massironi, Giacomo Mulinacci, Camilla Gallo, Alessandra Elvevi, Silvio Danese, Pietro Invernizzi, Edoardo Vespa

**Affiliations:** 1 Division of Gastroenterology, Center for Autoimmune Liver Diseases, European Reference Network on Hepatological Diseases (ERN RARE-LIVER), Fondazione IRCCS San Gerardo dei Tintori, 20900 Monza, Italy; g.mulinacci@campus.unimib.it (G.M.); c.gallo19@campus.unimib.it (C.G.); alessandra.elvevi@irccs-sangerardo.it (A.E.);; 2 School of Medicine and Surgery, University of Milano-Bicocca, 20125 Milan, Italy; 3 Gastroenterology and Endoscopy, IRCCS Ospedale San Raffaele, Vita-Salute San Raffaele University, 20132 Milan, Italy

**Keywords:** eosinophilic esophagitis, pathogenesis, soluble inflammatory mediators, Th2 response, tissue remodeling, cytokines, immune cells

## Abstract

Eosinophilic esophagitis (EoE) is a chronic inflammatory disease characterized by eosinophilic infiltration of the esophagus. It arises from a complex interplay of genetic predisposition (susceptibility loci), environmental triggers (allergens and dietary antigens), and a dysregulated immune response, mainly mediated by type 2 T helper cell (Th2)-released cytokines, such as interleukin (IL)-4, IL-5, and IL-13. These cytokines control eosinophil recruitment and activation as well as tissue remodeling, contributing to the characteristic features of EoE. The pathogenesis of EoE includes epithelial barrier dysfunction, mast cell activation, eosinophil degranulation, and fibrosis. Epithelial barrier dysfunction allows allergen penetration and promotes immune cell infiltration, thereby perpetuating the inflammatory response. Mast cells release proinflammatory mediators and promote eosinophil recruitment and the release of cytotoxic proteins and cytokines, causing tissue damage and remodeling. Prolonged inflammation can lead to fibrosis, resulting in long-term complications such as strictures and dysmotility. Current treatment options for EoE are limited and mainly focus on dietary changes, proton-pump inhibitors, and topical corticosteroids. Novel therapies targeting key inflammatory pathways, such as monoclonal antibodies against IL-4, IL-5, and IL-13, are emerging in clinical trials. A deeper understanding of the complex pathogenetic mechanisms behind EoE will contribute to the development of more effective and personalized therapeutic strategies.

## 1. Introduction

Eosinophilic esophagitis (EoE) is a chronic immune-mediated disease characterized by esophageal inflammation and dysfunction triggered by an abnormal Th2 inflammatory response [1,2,3,4,5]. The central pathogenesis of EoE revolves around a dysregulated immune response triggered by exposure to allergens, often derived from food [2,6]. This immune response leads to inflammation and tissue damage in the esophagus, resulting in the characteristic symptoms and histologic changes seen in EoE patients. Key elements of its central pathogenesis include allergen sensitization, Th2 immune response, epithelial barrier dysfunction, eosinophil infiltration, fibrosis, and remodeling [2,4]. In addition, genetic factors and environmental triggers play pivotal roles in the genesis of the disease.

Individuals with EoE develop sensitization to certain food antigens or environmental allergens [7]. This sensitization involves the activation of immune cells, particularly Th2 cells, which play a central role in driving allergic responses. Sensitized Th2 cells release cytokines, including interleukin (IL)-4, IL-5, and IL-13, which trigger an exaggerated immune response [5]. These cytokines promote eosinophil infiltration, mast cell activation, and tissue remodeling in the esophagus. Eosinophils release cytotoxic proteins and other inflammatory mediators that contribute to tissue damage and inflammation. In addition, the epithelial barrier of the esophagus is compromised in EoE, allowing allergens to penetrate the tissue more easily. Epithelial cells also exhibit altered gene expression, such as the downregulation of genes involved in differentiation and junctional proteins, resulting in the disruption of esophageal mucosal integrity.

Persistent inflammation and tissue damage trigger fibrosis and the remodeling of esophageal tissue. This leads to structural changes, such as the narrowing of the esophagus (strictures) and reduced esophageal compliance, mainly resulting in dysphagia [8].

Genetic susceptibility may play a role in the development of EoE, as certain genetic variations have been associated with an increased risk for the disease [9,10]. However, the exact genetic factors that contribute to susceptibility to EoE are still under investigation [10]. In addition to genetic predisposition, environmental factors such as diet and exposure to allergens or environmental allergens are also thought to influence the development and exacerbation of EoE. Certain foods, such as milk, wheat, and eggs, are often cited as triggers [11].

Understanding the central pathogenesis of EoE is critical to develop targeted therapeutic strategies that can reduce inflammation, restore esophageal function, and relieve symptoms [12]. However, the complexity of the disease is underscored by the involvement of multiple cellular and molecular pathways, making the development of effective treatments a multifaceted challenge.

Standard treatment therapies include dietary modification [11,13], esophageal dilation, and pharmacologic therapy [13,14]. Effective pharmacologic therapies include corticosteroids, rapidly evolving biological therapies, and proton-pump inhibitors (PPIs) [15]. A variety of novel therapeutic strategies have been developed targeting different cellular and soluble mediators that contribute to the complex mechanisms of the disease [16]. The pursuit of tailored approaches promises to improve patient outcomes and usher in a new era of precision medicine for people struggling with this complicated disease.

The aim of this review is to comprehensively explore the central pathogenesis, genetic and environmental factors, and therapeutic strategies associated with EoE. 

### Literature Search

References for this review were identified through searches of PubMed, Embase, and the Cochrane Library, with the search terms “Eosinophilic Esophagitis”, “Esophagus”, and “esophagitis” combined with the terms “pathogenesis”, “soluble inflammatory mediators”, “Th2 response”, “tissue remodeling”, cytokines”, and “immune cells” until June 2023. Articles were also identified through searches of the authors’ files. Only papers published in English were reviewed. The final reference list was generated based on originality and relevance to the broad scope of this paper.

## 2. Pathogenesis of Eosinophilic Esophagitis (EoE)

The pathogenesis of EoE involves a multifaceted interplay between genetic predisposition and environmental triggers, leading to a dysregulated immune response.

### 2.1. Genetic and Environmental Factors and Autoimmunity

EoE has a strong genetic component, as evidenced by various susceptibility loci identified through genetic studies. First-degree relatives of EoE patients have a 10 to 64 times higher risk of developing the condition compared with the general population [17,18], with an incidence among siblings of 2.4% vs. 5.5 per 10.000 in the general population [1,12]. Studies on twins reported an EoE frequency of 41% in monozygotic twins and 24% in dizygotic twins [18].

Moreover, certain genes, including TSLP, calpain-14 (CAPN14), Krüppel-like factor 13 (KLF13), and EMSY, have been associated with an increased risk of EoE [17,19,20,21]. Notably, the interplay between genes such as CAPN14 and specific environmental exposures highlights the complexity of EoE’s genetic–environmental interaction [1,22]. In particular, an association was found between the overexpression of CAPN14 in the early postnatal period of life (during breastfeeding and hospitalization in an intensive care unit) and exposure to certain environmental factors [12]. Overall, genetic risk factors are neither necessary nor sufficient to favor EoE, although they clearly modulate the individual lifetime risk of a given carrier.

Environmental factors such as diet, cesarean births, antibiotic use, formula feeding [9], PPI use during childhood, cold climates, indoor pollutants, and housing [10] have been linked to an increased EoE risk. Conversely, owning a furry pet in childhood and Helicobacter pylori infection are associated with a lower risk of EoE [10,12,23].

The role of the gut microbiota in immune regulation is increasingly recognized [24]. Exploring therapies that modulate the composition of the microbiota to mitigate inflammation and allergen sensitization holds potential for the treatment of EoE [25,26,27,28,29]. Specifically, patients with active EoE showed an increase in *Haemophilus* and *Aggregatibacter* species and a decrease in *Firmicutes* [30], and early interactions between epithelial cells and the esophageal microbiota were able to modulate CXCL16 expression and recruit invariant natural killer T cells to the esophageal epithelium [31]. In a study in mice, administration of *Lactococcus lactis* NCC 2287 resulted in the histologic remission of EoE [32], although further research is needed in this area.

The association with autoimmunity is increasingly being investigated, as 6% of patients with EoE have concomitant psoriasis, psoriatic arthritis, rheumatoid arthritis, or Hashimoto’s thyroiditis [33]. Indeed, antibodies against transmembrane desmoglein-3 (DSG3) and collagen XVII (NC16A) appeared to be increased in the sera of EoE patients [34].

### 2.2. Soluble Inflammatory Mediators of EoE

After allergen-mediated epithelial injury in the esophageal mucosa, several damage-associated molecular patterns (DAMPs) are released, contributing to the onset of a type 2 inflammatory response, a hallmark of EoE. Notable DAMPs include thymic stromal lymphopoietin (TSLP), interleukin (IL)-33, and IL-25. Their selective inhibition has been shown to prevent the development of specific food allergies [35]. DAMPs were among the first patterns to be recognized as molecules able to activate group 2 innate lymphoid cells (ILC2s), early effectors of type 2 mucosal immunity.

TSLP, an epithelial-derived cytokine expressed by several cell types, promotes Th2-type immune responses by influencing dendritic cells (DCs) [36]. Overexpression of TSLP is observed in the esophageal mucosa of EoE patients [37], and specific TSLP gene polymorphisms strongly correlate with the development of EoE in children [38]. TSLP signaling promotes dendritic cell activation, and the differentiation of naïve CD4 T cells into T helper 2 (Th2) cells and their proliferation by inducing IL-4 gene transcription, and supports immunoglobulin (Ig) E production [39,40]. A TSLP-triggered basophil-dependent mouse model of EoE-like disease was developed, and neutralization of TSLP ameliorated the disease [37].

IL-33, which is stored in the nuclei of various cells, including intestinal epithelial cells, is released upon exposure to environmental antigens [41,42]. Its binding to the ST2 receptor on immune cells, including eosinophils and mast cells, triggers a type 2 inflammatory response through the expression of costimulatory molecules such as CD86, major histocompatibility complex class II (MHC-II), and IL-6 [43,44,45]. IL-33 contributes to the expression of epithelial adhesion molecules, the release of cytokines and chemokines such as CXCL8 and CCL2, and eosinophil survival [46]. Increased levels of IL-33 have been described in the esophageal mucosa of patients with EoE [47], where it is expressed by both the endothelium and a subset of undifferentiated, nondividing esophageal epithelial cells in the basal layer [47,48]. Interestingly, a rare and novel chromosomal duplication of the entire IL-33 gene resulted in clinical features of EoE [49], whereas mouse models showed that the IL-33–ST2 axis is necessary to induce EoE [50] and to ensure homeostasis and the survival of eosinophils, as evidenced by reduced numbers of eosinophils in peripheral blood in IL-33- and ST2-deficient mice [51].

IL-25 belongs to the IL-17 cytokine family and is mainly expressed by Th2 cells and several epithelial cells. IL-25 activates immune cells and enhances IL-13 production by ILC2 in response to food allergens. It is produced in response to cell injury or tissue damage and activates immune cells through its interaction with the receptors IL-17RA and IL-17RB. IL-25 binding to the receptors activates several signaling pathways, such as NF-κB, MAPK, JAK, and STAT3, leading to several downstream effects such as inflammation and cell self-renewal, survival, or apoptosis [52]. Injection of exogenous IL-25 resulted in the proliferation of Th2 cells and ILC2 [53]. The effect of IL-25 on several allergy disorders has now been established [54]. As for EoE, the available data have shown that IL-25 is increased in active EoE compared with controls [55].

IL-4 and IL-13 play an important role in Th2 cell development. They are mainly expressed by basophils, eosinophils, mast cells, NK T cells, and ILC2 [56]. IL-4 and IL-13 share a common α-receptor, and they signal via the signal transducer and activator of transcription (STAT)-6, which allows them to exert specific functions in different cell types. They activate eosinophils and recruit them via CCL26 expression, regulate lymphocyte functions (Th2 differentiation and B cell IgG1 and IgE class switching) and macrophage M2 maturation, increase dendritic cell function [57], and promote epithelial barrier dysfunction by downregulating the epidermal differentiation complex (EDC) [58]. To date, IL-13 is considered the major effector cytokine in EoE, more abundant than IL-4 [57]. IL-13 actively induces the expression of VCAM-1/ICAM-1 in endothelial cells and IL-5 by lymphocytes, thereby promoting the migration of eosinophils from the bone marrow and inducing eosinophil homing to target organs [59]. IL-13 also acts on tissue remodeling by promoting epithelial cell hyperplasia, collagen deposition, and angiogenesis [60]. Lastly, its synergistic action with TGF-β1 can activate quiescent fibroblasts and differentiate them into myofibroblasts, and reduce the amplitude of esophageal muscle contraction [61].

Other inflammatory mediators such as IL-5, IL-18, IL-15, tumor necrosis factor alpha (TNF-α), the TNF-related cytokine LIGHT, and transforming growth factor β1 (TGF-β1) also contribute to EoE pathogenesis via different mechanisms. They modulate immune responses, eosinophil function, fibroblast activation, and tissue remodeling. IL-5 is expressed by Th2 lymphocytes, eosinophils, basophils, ILC2, CD34+ progenitor cells, and NKT cells. It induces the differentiation and maturation of eosinophils in bone marrow, homing in tissues, and protection from apoptosis [62,63].

Consequently, overexpression of IL-5 in patients with EoE is associated with disease activity and increased levels of eosinophils in the blood [64]. Of note, IL-5 also plays a role in basophil metabolism and function by causing them to increase histamine release upon activation [65]. IL-18 belongs to the IL-1 family, a pleiotropic cytokine produced mainly by macrophages, dendritic cells, and epithelial cells. Its overexpression has been noted in several atopic diseases, in which it exerts a pathological role by stimulating mast cell and basophil degranulation, recruiting granulocytes to the site of inflammation, inducing IgE production and isotype switching, and promoting a Th2 response [66,67]. It also induces invariant NK T cells to produce IL-5 and IL-13 [68]. Niranjanc et al. reported an increased concentration of IL-18 and its specific receptor IL-18Rα in the blood and esophageal mucosa of EoE patients, and this concentration correlated with the extent of esophageal eosinophilia, both in active and treated EoE patients [68].

IL-15 is predominantly produced by monocytes, macrophages, and dendritic cells. It stimulates the proliferation and differentiation of activated T cells and promotes the antigen-independent activation of NK cells [69]. IL-15 stimulates CD4+ T cells to produce the eosinophil-activating cytokines IL-5 and IL-13. It also induces esophageal epithelial cells to produce eosinophil-activating chemokines in mouse models and humans by expressing eotaxins-1–2 and eotaxin-3, respectively [70]. Tissue levels of IL-15 and IL-15Ra in the esophagus and blood levels of IL-15 were increased in patients with EoE compared with healthy individuals, and human IL-15 mRNA levels correlated with esophageal eosinophilia [64].

Tumor necrosis factor alpha (TNF-α) is an inflammatory cytokine with pleiotropic functions, among which it mediates the activation and survival of eosinophils. It is upregulated in EoE, particularly during active disease [71,72]. Upon esophageal inflammation due to external triggers, esophageal fibroblasts stimulate the release of TNF-α, which, in turn, promotes the epithelial-to-mesenchymal transition, fostering the development of esophageal fibrosis [73].

The TNF-related cytokine LIGHT is part of the TNF superfamily of cytokines that have emerged as important modulators of critical innate and adaptive immune responses. Recently, a major role of LIGHT in several eosinophilic disorders was described, including in EoE [74].

TGF-β1 is a pleiotropic cytokine involved in many different critical processes, including immune regulation, fibroblast activation, smooth muscle contraction, and the induction of the epithelial–mesenchymal transition. Patients with EoE have increased expression of TGF-β1 and its downstream nuclear transcription factor, phosphorylated SMAD2/3 protein [75]. Moreover, exposure to TGF-β1 correlates with a decreased expression of claudin-7, a tight junction membrane protein important for esophageal barrier function [76]. Consequently, chronic expression of TGF-β1 could allow allergen passage through the esophageal mucosa, leading to increased antigen presentation and immune activation. Nevertheless, there is conflicting evidence regarding the role of TGF-β1 in EoE, as some studies have shown that its mRNA levels are not increased in esophageal mucosa [77,78].

Eotaxins are a family of eosinophil-specific chemoattractant cytokines produced mainly by epithelial cells. They have been identified in several atopic diseases including EoE. They are mainly induced by IL-4 and IL-13, and they bind to the CCR3 receptor, which is predominantly expressed in eosinophils and mast cells [79], although they can also be released from activated eosinophils and mast cells. Notably, mice with a genetic deletion of CCR3 were protected from developing experimental EoE, demonstrating the role of eotaxins in the pathogenesis of the disease [71].

The eotaxin family includes eotaxins-1, -2, and -3, all of which are upregulated in EoE [80]. Among them, eotaxin-3 is the most abundant in EoE [71], and its expression is related to the concentrations of eosinophils and mast cells in esophageal biopsies [71].

Eotaxin-3 is encoded by the CCL26 gene, and the CCL26 concentration is able to distinguish EoE from healthy and GERD patients, respectively [80,81].

In contrast, the roles of eotaxins-1 and -2 in the pathogenesis of EoE appear to be marginal [72]. Indeed, deficiency in the former resulted in only modest attenuation of the disease [82,83], whereas the latter is mainly expressed in the lungs [84]. Finally, single-nucleotide polymorphisms in eotaxin-3 genes have been linked to EoE disease [85].

Although interferon-γ (IFNγ) is one of the major players in the type 1 inflammatory response, it has been reported to be increased in the mucosa of EoE patients [86]. In addition, an association with polymorphisms in a gene encoding a transcriptional regulator used by IFNγ has been described in patients with EoE [20]. Recently, in vitro treatment with IFNy was shown to increase esophageal barrier permeability and epithelial cell apoptosis [87], and EoE-causing allergens were able to stimulate CD4+ T cells to release IFNγ [86]. Overall, these data suggest a possible role of IFNγ in the pathogenesis of EoE, although further evidence is needed.

Immunoglobulin G4 (IgG4) is an unusually dynamic antibody with unique molecular features distinct from other IgG subclasses. Biopsy specimens from adult and pediatric patients with EoE showed the deposition of higher levels of food-specific IgG4 antibodies [88,89]. Notably, the symptoms and histopathology of IgG4 deposition disappeared after the avoidance of cow milk and reappeared after its reintroduction [90]. The mechanism underlying the role of IgG4 in EoE disease needs further investigation.

**Figure 1:**
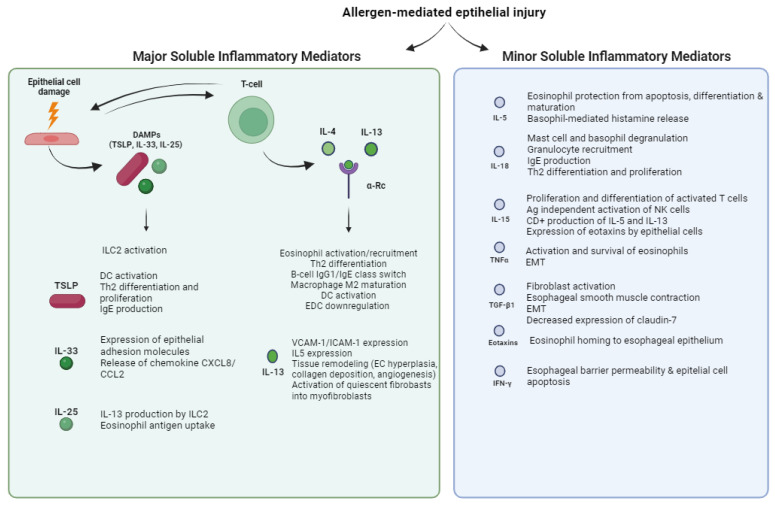
Functions of major and minor soluble inflammatory mediators of EoE. Allergen-mediated epithelial injury triggers the release of major and minor soluble inflammatory mediators, the former including damage-associated molecular patterns (DAMPs), IL-4, and IL-13, and the latter including IL-5, IL-18, IL-15, TNFα, TGF-β1, eotaxins, and IFNγ. TSLP = thymic stromal lymphopoietin; DC = dendritic cell; EC = epithelial cell; EMT = epithelial–mesenchymal transition; EDC = epidermal differentiation complex; α-Rc = α-Receptor.

A concise overview of each inflammatory mediator’s function, mechanisms, and implications in eosinophilic esophagitis (EoE) is resumed in Figure 1.

### 2.3. Cellular Mediators of EoE

Eosinophils are the hallmark of the pathogenesis of EoE, and their importance has been studied in both mouse and human models. These leucocytes were originally thought to be purely destructive end-stage effector cells because of their ability to release toxic granule proteins. However, recent research has revealed a more complex role in EoE pathogenesis. The recruitment of eosinophils through the release of inflammatory signaling molecules, such as alarmins and eotaxins, represents the early physiological response to mucosal injury by environmental antigens to maintain local mucosal homeostasis. Chronic exposure to stimuli and tissue damage leads to the release of mediators such as GM-CSF and IL-5, which promote the activation and local recruitment of eosinophils [91]. Furthermore, the release of eosinophil peroxidase, eosinophil cationic protein, and major binding protein directly leads to tissue damage and the dysfunction of vagal muscarinic M2 receptors, resulting in esophageal dysmotility [92]. Finally, eosinophils might also act as antigen-presenting cells [93]. Despite the pivotal role of eosinophils in disease pathogenesis, mouse models genetically engineered to lack eosinophils have shown that clinical features such as esophageal motility disorder are independent of eosinophil inflammation [94], and randomized controlled trials of anti-IL-5 therapies have failed to achieve clinical remission ([95]; NCT04543409). Overall, the data suggest that EoE is not entirely dependent on eosinophils and that broader targeting of type 2 immunity may be required.

Mastocytes are immune cells of the myeloid lineage traditionally associated with allergic reactions. They are classified according to whether they possess granules containing tryptase or tryptase and chymase, the latter being typical of esophageal mast cells both in diseases and under physiological conditions [96]. The trigger of mast cell activation in EoE is still unknown, as IgE-mediated mast-cell-dependent immediate responses to known food triggers have not yet been demonstrated, possibly due to the presence of non-IgE-mediated mechanisms [97]. Mast cells are major effectors in several atopic diseases, and their concentration and activation have been demonstrated in the esophageal mucosa of patients with EoE [94,98,99,100,101] and correlate with the extent of local infiltration of eosinophils [102] and EoE symptoms [96,103]. Mast cell degranulation, as detected by the presence of extracellular mast cell tryptase, was approximately 20-fold higher in EoE patients compared with control subjects [98]. Beyond their role in the type 2 inflammatory response, mast cells release specific mediators such as TGF-β1 tryptase, leukotrienes, prostaglandins, and histamine that contribute to esophageal smooth muscle hypertrophy and dysmotility [104,105].

Mast cells express the high-affinity receptor for IgE on their surfaces, which promotes a signal transduction process leading to the release of various inflammatory cytokines and chemokines [101]. Moreover, their ability to produce the eosinophil chemoattractant eotaxin-1 influences the accumulation of eosinophils in specific tissues [106], although some evidence for their ability to recruit eosinophils is still controversial [101]. Mast-cell-induced local inflammation of the esophagus results in altered mucosal permeability, smooth muscle hypertrophy, and altered contraction, leading to dysmotility [104,107]. Upon activation, mast cells promote tissue remodeling and fibrosis vis the expression of several profibrotic mediators such as TGF-β1 [108].

T cells play an important role in the pathogenesis of EoE, and several studies reported increased levels of T cells in EoE biopsies, with CD8 T cells predominating [109,110].

Antigen-mediated mucosal inflammation, the release of alarmins, and the production of cytokines lead to the activation of a Th2 response, which is responsible for initiating and maintaining non-IgE-mediated and type I IgE-mediated allergic reactions [111].

Th2 cells are responsible for the chemotaxis of eosinophils and for triggering the T2 cytokine cascade leading to the production of IL-13 and IL-5 [112].

Within the T cell family, regulatory T cells (Tregs) have the essential role of maintaining peripheral tolerance, preventing autoimmunity, and limiting chronic inflammatory diseases.

Decreased concentrations or altered functions of Tregs have been described in other atopic diseases [113], whereas their role in EoE pathogenesis is controversial, as their concentration has been reported to be either reduced [114] or increased [110,115] in mucosal samples. To date, none of the typical cytokines or interleukins associated with the Th2 response have been consistently and reproducibly altered to be used as a diagnostic tool for EoE. Compared with other atopic diseases (e.g., allergic asthma and atopic dermatitis), the lack of serological markers and unsatisfactory response to classic systemic immunosuppressive therapy in EoE may indeed reflect a different pathophysiology.

According to recent theories, EoE may be a local, autonomous Th2 disease with a unique pathogenetic pathway that relies exclusively on components of the esophageal mucosa (i.e., TSLP) [3].

An important function of T cells in EoE is related to their expression of the TNF-related cytokine LIGHT, which can induce an inflammatory phenotype in fibroblasts [70].

The role of B cells in the pathogenesis of EoE has been poorly studied because of their minimal infiltration of the esophageal mucosa compared with other immune cells [72,109]. On the other hand, Vicario et Al. not only confirmed the increased density of B cells in EoE but also demonstrated that biopsy specimens from the esophageal mucosa expressed germline transcripts, underscoring the potential of B cells to undergo local class-switch recombination [116].

Invariant natural killer T cells (iNKT) are a subset of T cells that when appropriately stimulated by sphingolipids (rather than protein antigens), can promote a Th2-type inflammatory response. Increased numbers of iNKT in the esophagus have been reported in EoE patients, particularly during active disease [116,117]. The iNKT concentrations in peripheral blood were inversely correlated with those in esophageal tissue samples, suggesting a local effect in the pathogenesis of EoE [116].

Interestingly, IL-13 induction of iNKT occurred only in EoE patients stimulated with cow-milk-derived sphingomyelin compared with α-galactosylceramide [116]. Thus, the recognition of specific milk-derived lipids and the resulting release of Th2-mediating cytokines may be a possible explanation for the role of iNKTs in the pathogenesis of EoE.

ILC2, an early effector of mucosal type 2 immunity, is lineage-negative since it lacks surface markers for T, B, natural killer (NK), and natural killer T (NKT) cells. It arises from lymphoid precursors in the bone marrow and is not antigen-specific [118], thus acting as a rapid, first line of defense before the activation of the adaptive immune response. Elevated ILC2 levels are found in various body regions, including the peripheral blood, gastrointestinal tract, lungs, nasal polyps, and skin. As in other Th2-mediated inflammatory diseases, DAMP-mediated activation of ILC2 led to its expansion and the secretion of high levels of type 2 inflammatory mediators such as IL-4, IL-5, IL-13, and amphiregulin [119,120]. The discovery of ILC2 has deepened the understanding of the type 2 immune response, and many studies have confirmed its dominance in typical type-2-mediated diseases such as asthma, atopic rhinitis, and atopic dermatitis [121]. Increased ILC2 levels have been detected in biopsies of esophageal mucosa from patients with active EoE compared with those with inactive EoE or control subjects, suggesting a role in disease pathogenesis [122]. However, the evidence is limited to a single study, and further studies are recommended.

Esophageal epithelial cells play a central role in the pathogenesis of EoE. Upon contact with inflammatory stimuli, they can express molecules of MHC-II and thus behave like non-professional antigen-presenting cells [123].

Under physiological conditions, the esophageal epithelium is relatively impermeable to medium- and large-size molecules, thus providing a barrier. In EoE, active inflammation results in damage to the epithelium associated with decreased expression of the structural proteins E-cadherin, desmoglein-1, involucrin, filaggrin, and synaptopodin [55,124], as well as the alteration of junctional proteins such as claudin and occludin [76,125]. The origin of these structural changes has been linked to the absence of the Kazal-type serine protease inhibitor (SPINK) 7, which is part of the differentiation program of the esophageal epithelium. SPINK7, which is highly expressed by healthy esophageal epithelial cells, was indeed significantly decreased in patients with EoE, who consequently exhibited higher permeability with dilated intercellular spaces [126,127]. Of note, the silencing of SPINK7 resulted in epithelial barrier dysfunction and activated transcriptional changes that stimulated the production of type 2 inflammatory responses [128].

**Figure 2:**
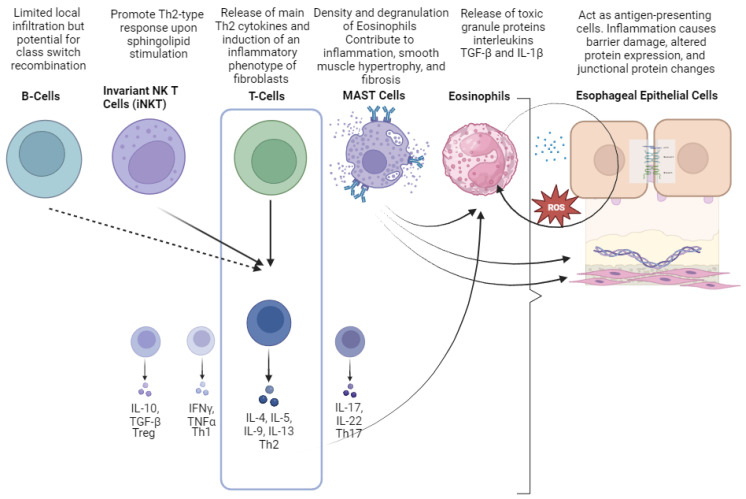
Cellular mediators involved in the pathogenesis of eosinophilic esophagitis (EoE). Different colors were used to represent and distinguish each cellular a soluble mediator.

Figure 2 summarizes the cellular mediators of EoE.

## 3. Therapies Targeting Pathophysiological Mechanisms

The therapeutic management of EoE is multifaceted and requires a multidisciplinary approach, incorporating evolving treatment options like proton-pump inhibitors (PPIs), corticosteroids, food elimination diets (FEDs), and targeted biological therapies. Table 1 summarizes the drugs targeting different mechanisms in the treatment of EoE.

### 3.1. Avoid Luminal Triggers

Based on the concept that EoE is a disease triggered by food antigens [141], a food elimination diet (FED) is now a recognized treatment alternative for EoE [129]. EoE patients exposed to cow’s milk fats exhibit increased production of IL-4 and IL-13 by esophageal T killer cells, which cause Th2-mediated local and systemic inflammation [116]. Elimination diets result in rapid clinical and histologic remission in up to 90% of cases [142], but adherence is a major concern due to taste, psychosocial impacts, and costs. Empirical elimination of common allergens such as cow’s milk protein, wheat, eggs, soy, peanuts, and fish/seafood results in a histologic response rate of approximately 50% in adults [130]. Sequential reintroduction of these allergens, followed by endoscopy and biopsies, identifies specific triggers. The stepwise approach, starting with the elimination of one allergen, provides better compliance and reduced nutritional risk [143]. Reduced FEDs targeting six (6-FED), four (4-FED), two (2-FED), or one (1-FED) allergens show similar response rates [144]. A meta-analysis confirmed comparable histological remission rates across diets [145]. Pediatric pilot studies are exploring the potential of epicutaneous immunotherapy in children with milk-induced eosinophilic esophagitis. This involves administering to children a specific type of cow’s milk containing Viaskin™ technology, which aims to deliver microgram amounts of allergens to the immune system through intact skin [146].

PPIs serve as an established first-line therapy for EoE [129]. By inhibiting gastric acid secretion, they decrease esophageal mucosal exposure, which disrupts the epithelial barrier [16]. PPIs also modulate chemotactic factors, reducing eosinophil attraction [147]. Eotaxin-3, a crucial EoE cytokine, is directly blocked by PPIs [148]. PPIs inhibit ICAM-1 and VCAM-1, thereby attenuating eosinophil expression and tissue remodeling [149].

### 3.2. Targeting Epithelial Barrier

The role of the altered esophageal epithelial barrier in EoE is extensively documented, yet the debate continues about whether these epithelial changes are a cause or a consequence of EoE [150]. Therapeutic strategies targeting the epithelial barrier have emerged, such as sucralfate, which enhances vascular flow and mucus production via prostaglandin and growth factors, offering cytoprotective effects against hydrogen-ion-mediated damage [151]. Clinical trials investigating these approaches are ongoing, such as a phase I trial (NCT0235307).

### 3.3. Targeting Soluble Mediators

#### 3.3.1. Corticosteroids

Corticosteroids modulate the NF-kB inflammatory cascade by targeting the nuclear factor kappa-light-chain-enhancer of activated B cells [152]. They directly decrease the expression of eotaxin-3, thereby reducing the recruitment of eosinophils [153]. Steroids suppress the production of eosinophils mediating the release of IL-13 in the esophagus via the JAKSTAT6 pathway, inhibiting inflammation and fibrosis [154]. Swallowed topical steroids such as fluticasone and budesonide reduce inflammation with minimal systemic absorption, ensuring safety and efficacy [129]. Orodispersible budesonide and orally administered viscous budesonide show efficacy [131,155].

#### 3.3.2. Drugs Targeting Interleukin (IL)-4/IL-13

Dupilumab is a fully humanized monoclonal antibody (mAb) that inhibits the common receptor component for IL-4 and IL-13, a key driver of EoE type 2 inflammation [9]. A phase II, double-blind, placebo-controlled trial showed that a 600 mg dupilumab loading dose followed by 300 mg weekly induced a significant clinical improvement, a remission rate of 82.6%, and a deep remission rate of 65.2% [132]. Similarly, a recent phase III randomized controlled trial (RCT) confirmed that subcutaneous administration of 300 mg of dupilumab relieved symptoms and improved histology, with statistically significant results compared with the placebo (60% and 5% histologic remission rates, respectively). No difference was observed between the weekly or every-2-weeks regimen [156]. The long-term effects of dupilumab on EoE are currently being evaluated in two phase III RCTs (NCT03633617; NCT04394351). Dupilumab has been approved by the FDA for >12-year-old and >40 kg EoE patients. In Europe, pre-marketing approval by the European Union for EoE is pending following a positive opinion by the Committee for Medicinal Products for Human Use.

Similarly, cendakimab and dectrekumab have demonstrated efficacy, while further studies are underway to determine their potential (NCT04753697).

#### 3.3.3. Drugs Targeting Other Interleukins (IL-5 and IL-15)

In mouse models, mucosal IL-5 overexpression can induce EoE, and IL-5 neutralization can almost completely prevent IL-13-induced EoE [82]. Mepolizumab and reslizumab are two monoclonal antibodies (mAb) against IL-5. Mepolizumab, a fully humanized anti-IL-5 mAb, showed improvement in symptoms, endoscopic features, and esophageal eosinophil counts but no histologic remission in several studies examining its efficacy in EoE [157,158]. A new multicenter, double-blind, placebo-controlled RCT with subcutaneously administered mepolizumab is currently ongoing (NCT03656380). Similarly, reslizumab, a humanized IgG4 kappa mAb that binds to human IL-5, showed a reduction in eosinophil infiltrate but little disease remission in a double-blind, placebo-controlled RCT in 226 EoE patients [159].

CALY-002 is the first-in-class humanized mAb inhibiting IL-15. An open-label cohort study focusing on EoE patients is currently ongoing as part of a multicenter, placebo RCT for celiac disease (NCT04593251).

#### 3.3.4. Drugs Targeting TGF-β and Prostaglandin Pathways

As explained in detail, TGF-β regulates epithelial growth and tissue remodeling, and, high levels of this factor have been repeatedly found in esophageal biopsies of active EoE patients [160]. Anti-TGF-β therapy could be used to prevent fibrosis and remodeling in EoE patients. Losartan, an indirect TGF-β inhibitor, shows potential in reducing fibrosis in human organs [161], with an ongoing phase II trial evaluating its effectiveness in EoE (NCT03029091).

As previously illustrated in detail, prostaglandins play an established role in the inflammatory response by recruiting inflammatory cells (mast cells, eosinophils, and basophils) by binding to the CRTH2 receptor. Timapiprant, a CRTH2 inhibitor, was studied in a double-blind, placebo-controlled RCT in 26 refractory EoE patients, and it demonstrated a significant reduction in symptoms and a decrease in eosinophilic infiltrate, but it did not achieve histologic remission [133]. On the other hand, montelukast, a competitive selective leukotriene receptor agonist, which proved effective for prostaglandin reduction in asthma, did not reach significant results in EoE, according to a cohort study [134].

#### 3.3.5. Drugs Targeting the IgE Pathway

EoE pathogenic mechanisms involve a non-IgE-mediated inflammatory response against food allergens; thus, humoral immunity does not play a crucial role in EoE’s pathogenesis [162], but increased esophageal IgE production has been described in EoE patients [163]. For this reason, omalizumab, a mAb that binds to free serum IgE and prevents it from binding to high-affinity receptors, has been investigated in EoE, but it showed disappointing results [135].

#### 3.3.6. Drugs Targeting Sphingosine-1-Phosphate (S1P), Integrin Pathways, and Sialic-Acid-Binding Ig-Like Lectins (Siglecs)

Selective sphingosine-1-phosphate (S1P) receptor modulators offer potential given their effects on eosinophil recruitment, the migration of NK cells, mast cell activation, lymphocyte trafficking, Th17 cell polarization, and dendritic cell differentiation [129]. A phase II-b double-blind, placebo-controlled RCT on the application of etrasimod in EoE is currently ongoing (NCT04682639).

Alpha4β7 integrin is overexpressed in EoE Th2 cells and, like in other Th2 inflammatory-cascade-mediated diseases, it promotes eosinophils and other inflammatory cells’ recruitment through its interaction with the MAdCAM-1 receptor [164]. Natalizumab, a humanized anti-α4β7 and -α4β1 mAb registered for multiple sclerosis, was reported to lead to the complete resolution of EoE that was not responsive to PPIs, swallowed corticosteroids, and a 6-FED in a woman treated for multiple sclerosis, with the persistence of clinical and histological remission after 3 years of treatment [136]. No further evidence is, to date, available. Likewise, vedolizumab, a selective anti-α4β7 mAb, proved to be an effective rescue therapy for eosinophilic gastroenteritis and was demonstrated to lead to deep esophageal histological remission in two cases of Crohn’s disease and concomitant EoE [137,165].

As for α4β7 integrin, Siglecs are also found on the membranes of many immune cells, and they are involved, among other cell signaling pathways, in apoptosis [166]. Siglec-8 blockers (antolimab/lirentelimab mAb), which were originally applied for eosinophilic enteritis, provided a significant reduction in esophageal eosinophilic infiltrate with a high esophageal remission rate and a dysphagia score reduction in a subcohort of EoE patients [167]. A phase II/III, multicenter, double-blind, placebo-controlled RCT study (NCT04322708) specifically in EoE is currently ongoing.

#### 3.3.7. Drugs Targeting JAK-STAT6 Pathway

Esophageal-recruited eosinophils secrete eotaxin-3, one of the most expressed cytokines in EoE patients, via the JAK-STAT6 pathway induced by the IL-4/IL-13-CCR3 interaction [58]. A single case report on the effectiveness of tofacitinib, a pan-JAK inhibitor, for EoE, is, to date, available. Further studies on the application of tofacitinib in EoE may be worth the effort [138].

### 3.4. Targeting Cellular Mediators

#### 3.4.1. Drugs Targeting Eosinophils

Benralizumab is a fully humanized anti-IL5-Rα antibody that inhibits the recruitment of inflammatory cells stimulated by IL-5 secreted by eosinophils [129]. The preliminary results from a double-blind, placebo-controlled study RCT (NCT03473977) evaluating the efficacy of benralizumab in patients with eosinophilic gastroenteritis report a significantly higher rate of histologic remission compared with the placebo but no significant clinical or endoscopic improvements. A phase III trial (NCT04543409) was prematurely terminated due to failure to achieve the co-primary endpoint of clinical remission, despite a significant reduction in eosinophil levels. Consequently, this underscores that eosinophils are not the exclusive therapeutic target, and their elimination alone is insufficient to ameliorate patient well-being.

#### 3.4.2. Drugs Targeting T Cells

The well-known purine analog azathioprine (or 6-mercaptopurine), which exerts its immunomodulatory effects through a cytotoxic action on inflammatory cellular players, thus inhibiting both cell-mediated and humoral immune responses, produced clinical and histological remission in steroid-refractory adult and pediatric EoE patients in some case reports [168]. However, solid evidence of its efficacy in EoE is still lacking.

BT-11, an orally administered, gut-restricted small molecule that activates lanthionine synthetase C-like 2 (LANCL2) and that has been shown to increase the number and function of Tregs in IBD [169], is being tested in a phase Ib study specifically in EoE patients (NCT04835168).

#### 3.4.3. Drugs Targeting Mast Cells

Cromolyn sodium, an inhibitor of mast cell degranulation by suppressing the transmembrane influx of calcium ions and, consequently, a suppressor of mast cell and eosinophil activation [170], has been tested as a therapeutic option for EoE, but neither clinical nor histologic effects have been reported [171]. Barzolvolimab is a humanized monoclonal antibody that specifically binds to the receptor tyrosine kinase KIT, reducing mast cell degranulation and mast cell numbers, and will be evaluated for its efficacy and safety in adult EoE patients in an upcoming study (NCT05774184).

#### 3.4.4. Immunotherapy for Environmental Allergens

As previously reported, not only food allergens but also environmental allergens may play a causative role in EoE’s pathogenesis. This theory is based on the apparent association between EoE and concurrent asthma/rhinoconjunctivitis and seasonal exacerbations of EoE [172,173]. Indeed, subcutaneous immunotherapy for environmental allergens has shown histologic remission of EoE in some case reports/small series [139,140].

## 4. Future Perspectives in EoE Targeted Therapy

As our understanding of EoE continues to deepen, novel therapeutic approaches are emerging, offering hope for improved management of this complex disease. However, the field also faces several challenges that need to be addressed to effectively treat EoE and enhance patients’ quality of life.

As mentioned previously, the underlying factors driving esophageal epithelial changes in EoE are the epidermal differentiation complex (EDC), a cluster of genes responsible for epithelial differentiation, and calpain (CAPN)-14, an intracellular regulatory protease. Downregulation of EDC leads to decreased expression of key proteins such as filaggrin (FLG), involucrin, E-cadherin, and other junctional proteins such as desmoglein-1 and zonulin-1. Similarly, overexpression of CAPN14 and other serine protease inhibitors such as serine proteinase inhibitors (SERPINs) and serine protease inhibitors of the Kazal type (SPINKs) contributes to the decreased presence of junctional proteins in EoE patients [130].

Short-chain fatty acids (SCFAs) such as butyrate and propionate have the potential to downregulate CAPN14 expression and increase the expression of FLG and other junctional proteins in vitro, leading to improved esophageal epithelial barrier integrity [174]. However, in vivo studies have not yet been performed to confirm these findings. Calpeptin, a potent protease inhibitor, has shown inhibition of CAPN14 in asthma mouse models [175], suggesting its potential therapeutic role in EoE.

IL-33, a key factor in triggering Th2 inflammatory responses, represents an attractive target for the treatment of EoE. Etokimab [176] and astegolimab [177], both monoclonal antibodies that selectively inhibit the IL-33–ST2 axis, are expected to show promising results in EoE therapy. However, there are no specific studies focused on EoE yet.

IL-18 inhibitors, including tadekinig alfa, a recombinant IL-18-binding protein [178], and GSK1070806, a humanized antibody targeting IL-18 [179], have shown success in other inflammatory diseases. These inhibitors are suggested as potential therapeutics for EoE, although specific studies are needed.Targeting TSLP, which plays a role in reducing esophageal eosinophilia, has led to FDA approval of tezepelumab, a human anti-TSLP monoclonal antibody, for experimental use in EoE [180]. Eotaxin-3 is a key target for a variety of EoE therapies. Novel small molecules such as GW766994 [181] and R321 [182] are promising eotaxin antagonists and may have potential for the treatment of EoE.

Inhibition of the JAKSTAT6 pathway as an alternative to tofacitinib (using agents such as ruxolitinib and leflunomide) may be a viable option to suppress IL-13-induced eotaxin-3 expression in esophageal epithelial cells, as suggested by in vitro studies [183,184].

Antihistamines have not been studied specifically for EoE, but the high expression of the histamine receptors HR1, HR2, and HR4 in the esophagus of active EoE patients [185] suggests their potential as an alternative therapeutic approach.

Concerning therapies targeting cellular mediators, dexpramipexole, a dopamine agonist, represents an interesting strategy to inhibit eosinophil maturation and could be explored for the treatment of EoE [186].

Targeting iNKT cells with humanized anti-CD1d and anti-Va24Ja18 antibodies has also shown promise in mouse models [187], but no human studies are yet available.

Proteomics analysis of esophageal mucosa may reveal key features of the pathophysiology of eosinophilic esophagitis, as the measurement of RNA and proteins in esophageal biopsies could provide essential information to fully understand the molecular mechanisms of EoE [188].

In summary, the landscape of EoE therapy is rapidly evolving, opening promising avenues for more effective and personalized treatments. Collaborative efforts by clinicians and researchers and continued investment in research, clinical trials, and patient-centered care will help to address challenges and make advances in the treatment of EoE.

## 5. Conclusions

Eosinophilic esophagitis (EoE) is a complex disease with increasing incidence and prevalence worldwide. The exact pathogenetic mechanism remains elusive, but certain environmental factors may trigger inflammation in the esophageal mucosa in genetically predisposed individuals. This, in turn, leads to the release and activation of numerous cellular and soluble mediators, ultimately resulting in a type 2 inflammatory response. Current treatment options include exclusion diets, proton-pump inhibitors, topical and oral corticosteroids, and the recently approved biologic anti-IL-4 agent dupilumab. The integration of personalized medicine approaches, including biomarkers, proteomic, transcriptomic, and genomic profiling, holds promise for describing the disease phenotype and facilitating the development of tailored treatment strategies. Understanding the underlined disease mechanisms could help target different pathophysiological moments for more effective treatment.

## Figures and Tables

**Table 1:** Drugs targeting different mechanisms in the treatment of eosinophilic esophagitis (EoE).

Type of Therapy	Specific Therapeutic Intervention/Drug	Specific Pathophysiological Target Mechanism	Clinical Evidence and Remarks
Avoiding luminal Triggers	Food elimination diet (FED)—eliminating from 1 up to 6 food allergens (from 1-FED to 6-FED)	Elimination of food antigens to reduce inflammation	Alternative second-line therapy [129]
	Empirical allergen elimination	Elimination of common allergens to identify triggers	50% proved efficacy—suggested sequential reintroduction [130]
	Proton-pump inhibitors (PPIs)	Inhibition of gastric acid secretion and esophageal barrier restoration	Established first-line therapy [129]
Targeting epithelial barrier	Sucralfate	Enhancement of vascular flow, mucus production, and cytoprotective effects	Ongoing phase I trial (NCT0235307)
Targeting soluble mediators	Corticosteroids	Modulation of NF-kB cascade and reduction in eosinophil and cytokines recruitment	Swallowed topical steroids are agreed second-line therapy with proven efficacy [131]
	Dupilumab, cendakimab, and dectrekumab	Inhibition of IL-4/IL-13	Dupilumab approved by the FDA for >12-year-old and >40 kg EoE patients with proven efficacy [132]. Long-term phase III trials for dupilumab are ongoing (NCT03633617 and NCT04394351). Phase III trials for cendakimab are also ongoing (NCT04753697).
	Mepolizumab and reslizumab	Neutralization of IL-5	Ongoing phase II trial for mepolizumab (NCT03656380).
	CALY-002	Inhibition of IL-15	Ongoing phase I trial (NCT04593251).
	Losartan	Indirect inhibition of TGF-β pathway	Ongoing phase II trial (NCT03029091).
	Timapiprant and montelukast	Inhibition of prostaglandins	Clinical efficacy but no histologic remission with timapiprant [133]; no clinical benefits of montelukast [134].
	Omalizumab	Inhibition of IgE pathway	Disappointing results in pilot study [135].
	S1P and integrin pathways	Modulation of S1P receptor and inhibition of α4β7 integrin	Ongoing phase II trials for S1P receptor modulator (NCT04682639). Proven efficacy of anti-α4β7 integrin [136,137].
	Antolimab and lirentelimab	Siglec inhibition: reduction in the esophageal eosinophilic infiltrate and inhibition of eosinophil secretion of eotaxin	Ongoing phase II/III trial (NCT04322708).
	Tofacinib	JAK-STAT6 inhibition	Single case report [138].
Targeting cellular mediators	Benralizumab	Inhibition of IL-5 receptor	No clinical benefits: phase III trial (NCT04543409) prematurely terminated, but preliminary results of histological remission in an ongoing phase III trial (NCT03473977).
	Azathioprine and BT-11	T cells	BT-11 ongoing phase I trial (NCT04835168)
	Cromolyn sodium and barzolvolimab	Mast cells	Ongoing phase II trial of barzolvolimab (NCT05774184).
	Immunotherapy for environmental allergens	Subcutaneous immunotherapy for environmental allergens	Histologic remission in small case reports/small series [139,140].

## Data Availability

Not applicable.
